# Effect of Degradation Intensity on Grassland Ecosystem Services in the Alpine Region of Qinghai-Tibetan Plateau, China

**DOI:** 10.1371/journal.pone.0058432

**Published:** 2013-03-04

**Authors:** Lu Wen, Shikui Dong, Yuanyuan Li, Xiaoyan Li, Jianjun Shi, Yanlong Wang, Demei Liu, Yushou Ma

**Affiliations:** 1 State Key Laboratory of Water Environment Simulation, Environmental School of Beijing Normal University, Beijing, China; 2 Qinghai Academy of Animal Science and Veterinary Medicine of Qinghai University, Xining, China; 3 Northwest Institute of Plateau Biology, Chinese Academy of Sciences, Xining, China; NASA Jet Propulsion Laboratory, United States of America

## Abstract

The deterioration of alpine grassland has great impact on ecosystem services in the alpine region of Qinghai-Tibetan Plateau. However, the effect of grassland degradation on ecosystem services and the consequence of grassland deterioration on economic loss still remains a mystery. So, in this study, we assessed four types of ecosystem services following the Millennium Ecosystem Assessment classification, along a degradation gradient. Five sites of alpine grassland at different levels of degradation were investigated in Guoluo Prefecture of Qinghai Province, China. The species composition, aboveground biomass, soil total organic carbon (TOC), and soil total nitrogen (TN) were tested to evaluate major ecological services of the alpine grassland. We estimated the value of primary production, carbon storage, nitrogen recycling, and plant diversity. The results show the ecosystem services of alpine grassland varied along the degradation gradient. The ecosystem services of degraded grassland (moderate, heavy and severe) were all significantly lower than non-degraded grassland. Interestingly, the lightly degraded grassland provided more economic benefit from carbon maintenance and nutrient sequestration compared to non-degraded. Due to the destruction of the alpine grassland, the economic loss associated with decrease of biomass in 2008 was $198/ha. Until 2008, the economic loss caused by carbon emissions and nitrogen loss on severely degraded grassland was up to $8 033/ha and $13 315/ha, respectively. Urgent actions are required to maintain or promote the ecosystem services of alpine grassland in the Qinghai-Tibetan Plateau.

## Introduction

Qinghai-Tibet Plateau (QTP), the so-called third pole of the world, is an important eco-region of the earth. In this region, more than 85% is covered by alpine grasslands [1]. Alpine grassland on the QTP provides great ecosystem services, such as plant diversity conservation, carbon sequestration, soil and water protection, as well as Tibetan culture and the maintenance of traditions, etc. [2]. As one of the major pastoral production bases in China, it supported 30 million sheep (including goats) and 12 million yaks in 2005 [3]. The soil holds more than twice as much carbon as can be found in its vegetation or the atmosphere [4], the changes in its soil carbon concentration can have a large effect on the global carbon budget [5]. Carbon and nitrogen in soil can not only determine soil quality, but also influence ecosystem productivity. As soil at high latitudes is expected to respond sensitively to climate change [6], alpine grassland ecosystems are considered influential in global environmental change, through carbon and nitrogen sequestration [7]. Grassland is also one of the important alpine genetic pools. It is thought that high biodiversity increases the stability of most types of ecosystems [8] and enhances the sustainability of resource exploitation [9]. However, it was reported that almost 30% of alpine grasslands were severely degraded due to the integrated effects of climate change, population increases, overgrazing and rodent (plateau pika, *Ochotona curzoniae*) damage [10]. Grassland degradation affected not only the livelihood of pastoralists, but also others who suffer from resultant hydrological disturbances, dust storms, commodity scarcity, and the social consequences of uprooted people [10], [11]. The ecosystem services of alpine grassland changed significantly with the increase of grassland degradation intensity. Understanding ecosystems from the perspective of humans as beneficiaries has tremendous potential for protecting ecosystems and the services they provided [12]. The process of identifying or evaluating ecosystem services is a powerful lens through which to understand human relationships with the environment and to design environmental policy [12]. So, it is necessary to access the economic loss or benefit caused by the change in ecosystem services.

Though some scholars around the world have done some research on assessments of degraded ecosystems, few of them quantified the dynamics of ecosystem services along a degradation gradient. Lange and Jiddawi [13] evaluated the ecosystem services of a degraded marine ecosystem that was caused by uncontrolled tourism development, rapid population growth, destructive fishing, overharvesting of mangroves, dumping of untreated wastewater from urban areas and periodic coral bleaching. However, whether the decline in ecosystem services changes under different disturbance intensities remains a question. Rounsevell et al. [14] provided a new framework that can be applied to investigate the complex dynamics of environmental change drivers to assess changes of ecosystem services. Tomback and Achuff [15] evaluated the values of white pines that suffered from blister rust, in terms of biodiversity, economics, ecosystem services and aesthetics. de Groot et al. [16] reviewed the challenges in ecosystem service assessment, and presented an integrated concept for assessing ecosystem services and values given land use and cover change. Ouétier et al. [17] studied the ecosystem service of mountain grassland in the French Alps associated with land use change. Although these studies have documented the service value of some degraded ecosystems and report methods and ideas to evaluate the services, little attention has been paid to the dynamics of ecosystem services with different degrees of ecosystem degradation.

For the alpine grassland of the QTP, many researchers have carried out the valuation of ecosystem services by using remote sensing data, geographic information systems (GIS), and simulation models [18]–[23]. However, none of them have used first-hand data obtained from the field to estimate changes in ecosystem services, with respect to the degree of grassland degradation. Therefore, in this study, we followed the Millennium Ecosystem Assessment classification scheme to choose four elements of ecosystem services (net primary productivity (NPP), carbon sequestration, nitrogen sequestration, and biodiversity maintenance) to evaluate the changes in ecosystem services shown in field data with respect to degree of degradation on alpine grasslands of the QTP. We hypothesized that ecosystem services decreased with the increase in grassland degradation intensity. The aim of this study is to provide the scientific foundation for natural ecological compensation or payment for environmental services (PES). Such efforts can also contribute to the debate concerning the achievement of sustainable development of alpine grassland in the QTP of China and worldwide.

## Materials and Methods

### Research Area

The study site is located in Dawu Village, Maqin Country of Guoluo Tibetan Autonomous Prefecture, Qinghai Province, China. The average elevation of this area is 4200 m with typical continental climate. The annually average temperature is −0.6°C, the lowest temperature is −34.9°C. Annual accumulated temperatures above 0°C and 5°C are 1202.6°C and 865.0°C respectively. Annual precipitation is 513 mm, occurring mainly from May to September and annual evaporation is 1459 mm. Annual sunshine hours are 2571 h, and there is no reliably frost-free period. The soil is silt-clay, which is classified as alpine meadow soil according to the Chinese Soil Classification System. The vegetation of this alpine grassland was dominated by alpine meadows composed mainly of *Kobresia* spp., *Polygonum* spp. and *Poa* spps. [24]. Since the 1970s, researchers have done a series of experiments on restoration of degraded grasslands in this area. They established several 100 m×100 m demonstration plots for displaying different degraded grasslands.

### Research Design

In this study, vegetation composition, aboveground biomass, soil total organic carbon and total nitrogen were measured in study sites with different levels of degradation (none, light, moderate, heave and severe). Four major ecosystem services of the alpine grassland, including biodiversity conservation, carbon sequestration, nitrogen maintenance and primary production, were selected for evaluation in this study. The reasons for choosing these four ecosystem services are described below.

Alpine grasslands in the QTP are grazed by indigenous herbivores, such as yak and Tibetan sheep [25]. The alpine grasslands have served as the dominant pastures for Tibetan communities over a long history and are regarded as one of the major pastoral production bases in China [2], [26].

In pastoral ecosystems, the amount of aboveground biomass not only determines forage availability, which thus constrains herbivore carrying capacity [27], but also is an important component of the global carbon cycle [28]. Soil is the largest organic carbon reservoir in the terrestrial biosphere, which is about two times larger than that of vegetation or the atmosphere [27]. Even a minor change in storage of organic carbon within soil could result in a significant alteration in atmospheric CO_2_ concentrations [28]. On the earth, the carbon storage of grasslands is 412∼820 million ton, accounting for 33–34% of the whole terrestrial ecosystem. The changing concentration of C and N within the soil can reflect not only soil quality and ecosystem productivity, but also the influence of C and N cycling and storage on global climate change [29]. As the QTP is one of the most sensitive areas to climate change [30], the changes of these services–carbon maintenance and nitrogen sequestration– play essential roles in global change.

The grassland in Qinghai-Tibetan Plateau is also one of the earth’s good alpine genetic pools. It has been reported that 15%–23% of indigenous plant species are endangered due to the degradation of the alpine grasslands, especially wet meadows which are key habitats for many alpine organisms in these headwater areas [31]. Thus understanding the states of biodiversity maintenance is critical for evaluating the services of the alpine grassland in QTP.

### Field Survey and Sampling

The parameters for grassland degradation and ecosystem services were measured in the field during growing seasons from July to August of 2008 and 2009. Vegetation composition, coverage and plant biomass were surveyed within four 100 cm×100 cm quadrats in each site of the alpine grassland at different degrees of degradation to quantify the level of grassland degradation in the present study. Plant diversity in each of degraded grasslands was surveyed in each site with thirty randomly distributed 50 cm×50 cm quadrats. Margalef richness index, Simpson index and Pielou evenness index were calculated.

Together with the vegetation surveys, five soil samples in each soil layers (0–10 cm, 10–20 cm, 20–30 cm) were collected with a soil auger (D  = 3.5 cm) from each quadrat. The soil samples were then pooled together, air-dried, and passed through 0.85 mm and 0.15 mm sieves for testing total organic carbon (TOC) and total nitrogen (TN). TOC was measured by a thermodilution method with potassium dichromate. TN was assayed by a Vario EI automatic elemental analyzer made by Elementar Company in Germany. The soil bulk density and organic carbon density (OCD) were calculated by Song et al.’s formula [32].

### Ecosystem Service Value Assessment

The evaluation in this study was a first attempt of integrating direct market valuation, indirect market valuation and contingent valuation to estimate the value of alpine grasslands along the degradation gradient based on the first-hand data from the field surveys. The assessment of the ecosystem service values of the alpine grassland in Qinghai-Tibet Plateau were standardized into US dollars ($1 =  RMByuan 6.85, August 2008), so as to reflect welfare in ways that are not mediated by the consumption of purchased goods. Because soil carbon, nitrogen and plant diversity losses did not all occur in a given year, the values for these services represent the values accumulated up to 2008.

Net primary production value V_NPP_ is estimated by the direct market price method as.
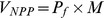
(1)Where P_f_ is the price of the forage production and M is the biomass of the forage. According to the price of the dry forage in the market in 2008, P_f_ is $80/t.

The value of ecosystem carbon sequestration (V_C_) is calculated by the carbon tax of Sweden ($150/Mg C).
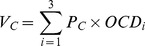
(2)Where P_C_ is the price of the Carbon tax, and OCD*_i_* is the organic carbon density of *i* ^th^ soil layer (Mg C/ha).

We applied the opportunity cost approach to assess the value of the nitrogen sequestration (V_N_) [33]. The average price of urea fertilizer was about $240/t in 2008.
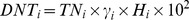
(3)

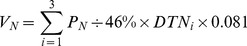
(4)Where, P_N_ is the price of the urea, 46% is the percentage of total N in urea (According to the data form Lin et al. [34]). DTN*_i_* is the density of total nitrogen of *i* ^th^ soil layer (Mg N/ha), TN*_i_* is the total nitrogen content in *i* ^th^ soil layer (%), 0.081 is the proportion of content of available nitrogen in total nitrogen, is soil bulk density of the *i* ^th^ soil layer, H*_i_* is soil thickness in the *i* ^th^ soil layer (cm), and *i*  = 1, 2, 3 represents soil layers at 0–10 cm, 10–20 cm, and 20–30 cm, respectively.

We categorized the value of biodiversity maintenance into 3 levels based on Margalef richness index [35]. When the Margalef richness index was less than 4.0, the value of biodiversity maintenance was $200/ha, when it was 4–4.5, the value is $300/ha, when it was more than 4.5, the value is $400/ha.

### Statistical Analysis

Differences among differently degraded pastures were analyzed with one-way ANOVA. Data were considered to be significantly different at *p*<0.05. The graph was draw using Origin 8.0 to present the data as the mean ± standard error. Due to limitations of current understanding, methods and data, there are potentially large margins of error associated with the estimates [36]. To price the ecosystem services, a variety of methods associated with direct market pricing, indirect market pricing, and replacement cost have been implemented.

## Results

### Primary Production/Aboveground Biomass

**Figure 1:**
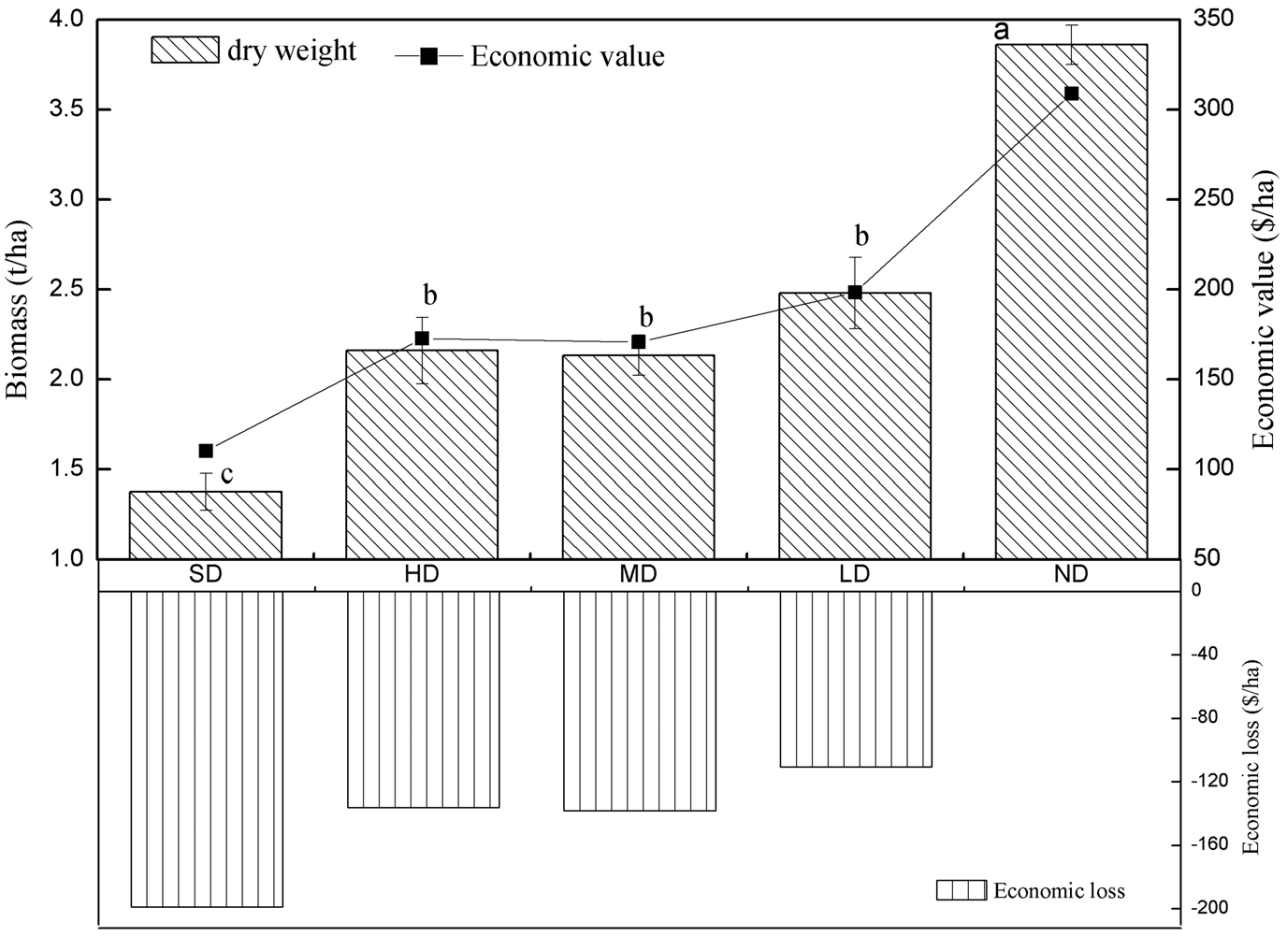
The Aboveground Biomass and Economic Loss of Different Degradation Grasslands. Note: ND, LD, MD, HD, SD represent non-degradation, light degradation, moderate degradation, heavy degradation and severely degradation, respectively.

Aboveground biomasses showed a decreasing trend with increasing pasture degradation (Figure 1). However, there was no significant difference between the biomass of heavily degraded grassland (dry weight 2.16±0.18 t/ha) and moderately degraded site (dry weight 2.13±0.11 t/ha) (*p*>0.05). At an average price of $80/t for dry grasses in 2008, when compared with the non-degradation grassland, the direct economic loss caused by light degradation reached $111/ha, and in severely degradation the direct economic loss was up to $198/ha.

### Soil Carbon Sequestration

**Figure 2:**
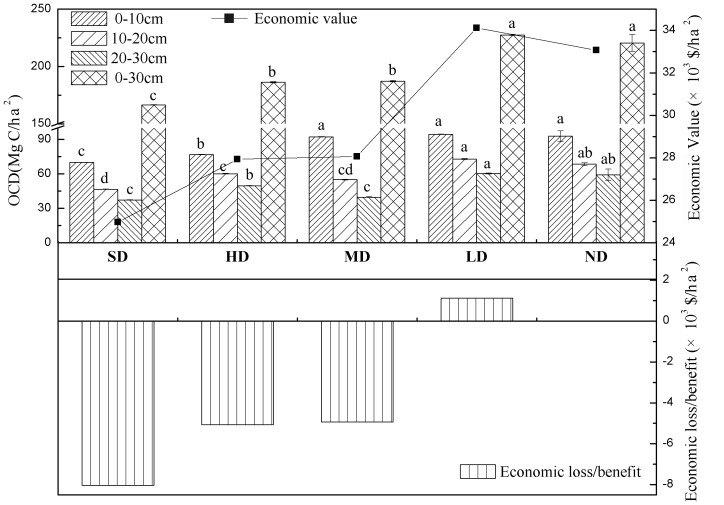
The Density of Soil Organic Carbon and the Economic Loss in Different Degradation Grasslands. Note: ND, LD, MD, HD, SD represent non-degradation, light degradation, moderate degradation, heavy degradation and severely degradation, respectively.

Along the grassland degradation gradient, the soil organic carbon density for each soil layer reached the maximum in light degradation grassland (Figure 2). The OCD of moderately, heavily and severely degraded grasslands were all lower than that of non-degraded grassland. The density of organic carbon decreased along the soil profile. The result shows that total organic carbon of the 0–30 cm soil layer increased at light degradation and decreased at moderate and severe degradation.

We used the density of organic carbon to reflect the ecosystem function of carbon sequestration. Using the carbon tax of Sweden ($150/Mg C) to calculate the value of CO_2_ emissions [23], the potential economic income caused by carbon sequestration in the first soil layer (0–30 cm) were $25.0×10^3^/ha, $27.9×10^3^/ha, $28.1×10^3^/ha for severely degradation, heavy degradation and moderate degradation, respectively. However, in lightly degraded grassland, the benefit gained from carbon sequestration reached $34.1×10^3^/ha, which is a little higher than non-degraded lands, at $33.1×10^3^/ha.

### Nitrogen Sequestration

**Figure 3:**
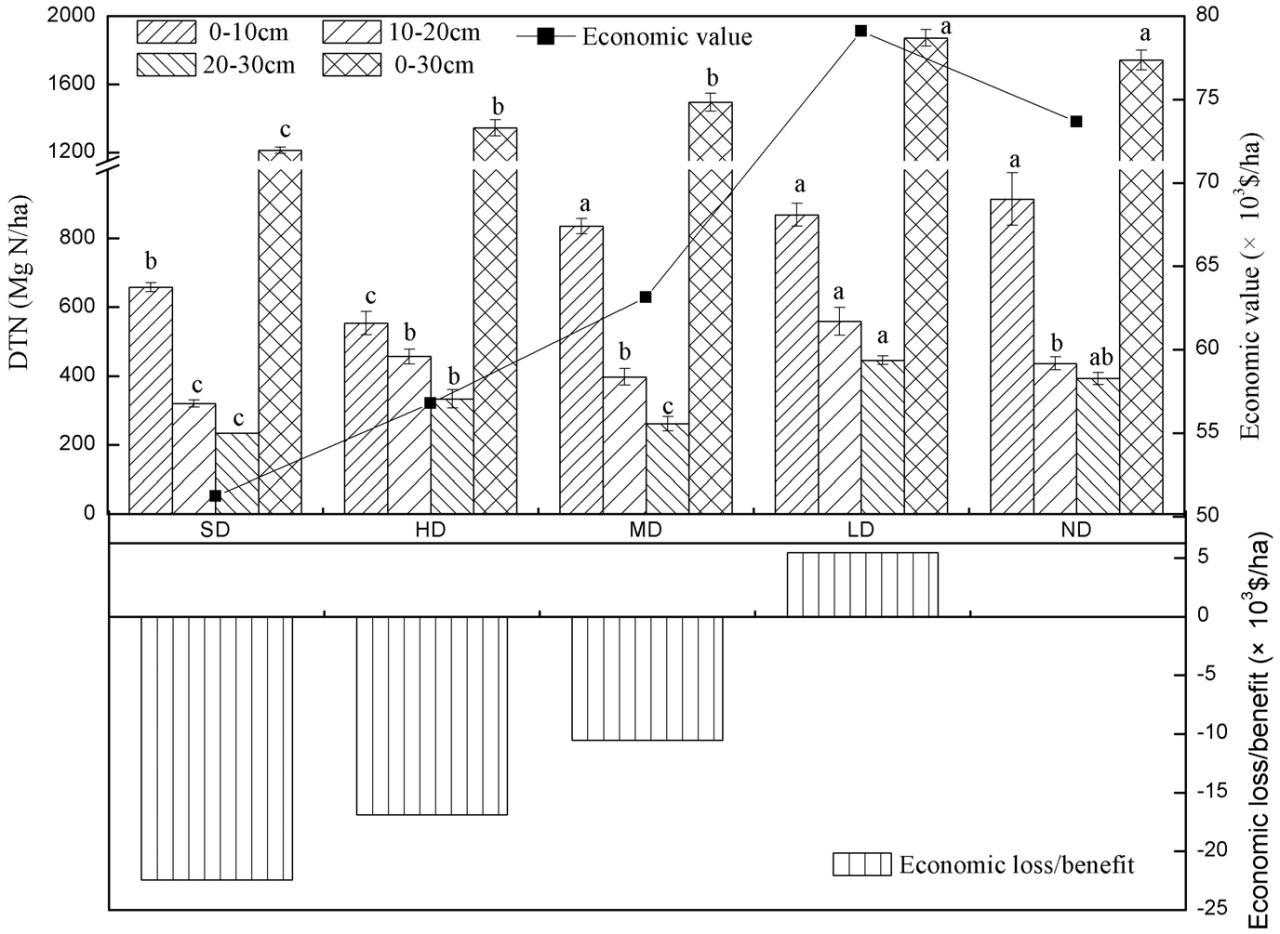
The Density of Soil Total Nitrogen and the Economic Loss of Different Degradation Grasslands. Note: ND, LD, MD, HD, SD represent non-degradation, light degradation, moderate degradation, heavy degradation and severely degradation, respectively.

Changes in soil total nitrogen along the degradation gradient are shown in Figure 3. The trend of DTN across soil layers was consistent with that of OCD. The economic benefit caused by nitrogen sequestration of severely, heavily, moderately, lightly, and non-degraded grasslands were $51.3×10^3^/ha, $57.4×10^3^/ha, $58.6×10^3^/ha, $69.9×10^3^/ha, and $64.6×10^3^/ha, respectively. Thus, compared to the non-degraded sites, both severely and heavily degraded grasslands showed serious economic declines due to nitrogen losses. Only in the light degradation site was the value of nitrogen sequestration higher than non-degraded grasslands.

### Plant Diversity Maintenance

**Figure 4:**
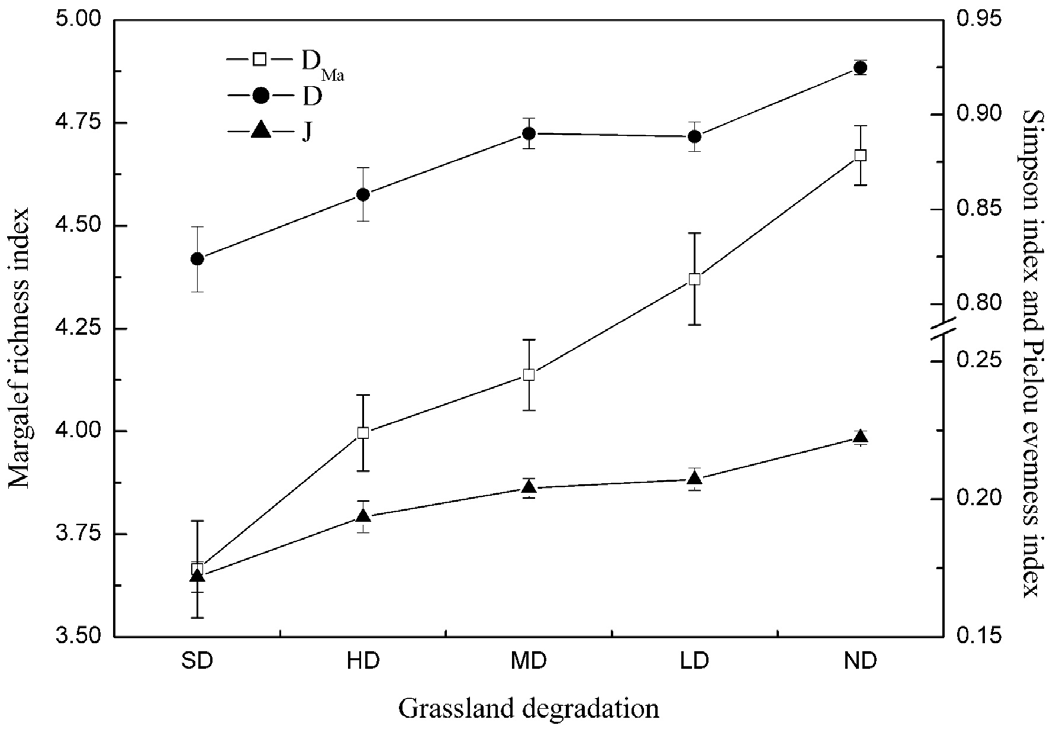
The Plant Diversity of Different Degradation Grasslands. Note: ND, LD, MD, HD, SD represent non-degradation, light degradation, moderate degradation, heavy degradation and severely degradation, respectively.

Figure 4 shows that the trends in the Margalef richness index, Simpson index and Pielou evenness index in grasslands degraded to different degrees were almost the same. The three plant diversity indexes in the non-degraded sites were the highest, and those in severely degraded sites were the lowest. Along the degradation gradient, the plant diversity indexes decreased, except the Simpson index in moderate degradation grasslands, which is higher than that of light degradation grassland.

The value of plant diversity maintenance in non-degradation grassland was $400/ha, and in light, moderate and heavy degradation grasslands were $300/ha, but in severely degradation it was only $200/ha.

## Discussion

The results of valuation for alpine grassland ecosystem show that the values of non-degraded grassland are $308.9/ha, $33.07×10^4^/ha, $64.58×10^4^/ha and $400/ha for the services of production provision, carbon sequestration, nitrogen sequestration and biodiversity maintenance, respectively (Table 1). NPP is a parameter used to quantify the net carbon absorption rate by living plants, and has been shown to be correlated with spatially fungible ecosystem services [36]. As the magnitude of grassland degradation increased, the biomass (fresh weight or dry weight) decreased. The trend in biomass observed is consistent with results of previous research on biomass [37]–[39]. The function of carbon sequestration shows a decreasing trend, possibly because the underground biomass decreases with increasing grassland degradation and soil depth [40]. The trend of carbon maintenance was consistent with previous research [41], in which it was found that TOC was higher in lightly degraded grassland than in non-degraded grassland. In this study, we conclude that the function of carbon maintenance and nitrogen sequestration have similar dynamics in alpine grassland. This conclusion is consistent with other scholars’ findings [42], [43]. The decrease of these two functions may be associated with human disturbances, as it was found that the structure and function of grassland ecosystems were affected by anthropogenic factors such as seasonal overgrazing [44]. Overgrazing not only altered the vegetation composition but also resulted in a gradual decrease of soil organic carbon and nitrogen in the alpine meadow ecosystem [45].

**Table 1:** The Total Economic Loss/Benefit of the Alpine Grassland Caused by Degradation.

Degradation	Area	Total economic loss/benefit in total area (×10^7^ $)			
intensity	(×10^7 ^ha)	NPP	Carbon sequestration	Nitrogen sequestration	Biodiversity maintenance
ND	0.19	0	0	0	0
LD	0.49	−54.17	549.54	2626.41	−49.00
MD and HD	0.97	−134.03	−4785.32	−5836.73	−97.00
SD	0.28	−55.66	−2249.26	−3728.27	−56.00
Total	1.93	−243.86	−8485.04	−6938.59	−202.00

Note: ND, LD, MD, HD, SD represent non-degradation, light degradation, moderate degradation, heavy degradation and severe degradation, respectively.

Our trend in the Simpson index was almost opposite to that of Zuo’s et al.’s [39] findings that there was an increasing trend in the Simpson index in the processes of grassland degradation. Wang et al. [46] reported that the plant diversity indices, including species richness, Shannon-winner index and Pielou evenness index were higher in light and moderate degradation stages than in the other stages of degradation. Some scholars also found that as degradation strengthened, plant diversity demonstrated different tendencies, with a decreasing trend or a trend shaped like a “humped-shaped response” [46].

It is clear that ecosystem services varied with degradation intensity. As grasslands are degraded, the direct economic loss caused by biomass decreasing reached $198/ha in severely degraded grassland. The indirect economic loss in the severely degraded grassland caused by carbon emission, nitrogen loss (0–30 cm) and plant diversity decrease were up to $8 033/ha, $13 315/ha, and $200/ha, respectively. Both heavily degraded and moderately degraded grasslands show financial losses caused by decreases in these services. However, the lightly degraded grassland gain some economic benefit compared to the non-degradation grassland caused by carbon maintenance and nitrogen sequestration. The value is higher than reported in Xie et al.’s [22] research, in which the value of alpine grassland services equaled $295/ha by using empirical formulas. Liu et al. [18] reached the result that service value of alpine grassland is $516.5/ha by using Xie’s [22] method according to data drawn from GIS.

According to the valuation of different degraded grasslands [47], in the Qinghai-Tibetan region the area of severely degraded is up to 2.829×10^6^ ha, and 9.648×10^6^ ha is moderately and heavily degraded. It can be inferred that in alpine regions, the retrogression of grasslands in 2008 dollars brought the economic loss of $2.44 billion by decreases in NPP. Because of the carbon emission, nitrogen loss and plant biodiversity loss, the alpine grassland had lost $84.85 billion, $69.39 billion and $2.02 billion up to 2008. However, this finical loss is only a small part of actual losses owing to the grassland destruction. In this study, we neglect the value of gross primary production (GPP), recreation value and the potential synergy among these ecosystem services. Regardless, urgent actions are needed to maintain or promote the ecosystem services of alpine rangelands in the Qinghai-Tibetan Plateau.
